# Sequence variation of PfEMP1-DBLα in association with rosette formation in *Plasmodium falciparum *isolates causing severe and uncomplicated malaria

**DOI:** 10.1186/1475-2875-8-184

**Published:** 2009-08-04

**Authors:** Natharinee Horata, Thareerat Kalambaheti, Alister Craig, Srisin Khusmith

**Affiliations:** 1 Department of Microbiology and Immunology, Faculty of Tropical Medicine, Mahidol University, 420/6 Rajvithi Road, Bangkok 10400, Thailand; 2 Liverpool School of Tropical Medicine, Pembroke Place, Liverpool L3 5QA, UK

## Abstract

**Background:**

Rosetting and cytoadherence of *Plasmodium falciparum-*infected red blood cells have been associated with severity of malaria. ICAM-1 and CD36 are the main host cell receptors, while PfEMP1-DBLα is a major parasite ligand, which can contribute to rosette formation. This study is aimed at demonstrating whether the highly polymorphic PfEMP1-DBLα sequences occurring among Thai isolates causing severe and uncomplicated malaria are associated with their ability to form rosettes and reflected the clinical outcome of the patients.

**Methods:**

Two hundred and ninety five PfEMP1-DBLα sequences from Thai clinical isolates causing severe and uncomplicated malaria were evaluated by sequencing and direct comparison using the specific text string analysis functions in Microsoft Excel and Perl. The relationships between the PfEMP1-DBLα sequences were also analysed by network analysis. The binding abilities of parasitized red blood cells (PRBCs) to CD36, wild type ICAM-1, ICAM-1^Kilifi ^and ICAM-1^S22/A ^under static condition were included.

**Results:**

Two hundred and eighty one non-identical amino acid sequences were identified (< 95% sequence identity). When the distributions of semi-conserved features (PoLV1–4 and sequence group) within the rosetting domain PfEMP1-DBLα were observed, close similarity was found between isolates from the two disease groups. The sequence group 1 representing uncomplicated malaria was significantly different from the sequence group 3 representing the majority of severe malaria (*p *= 0.027). By using a simple non-phylogenetic approach to visualize the sharing of polymorphic blocks (position specific polymorphic block, PSPB) and cys/PoLV among DBLα sequences, the sequence group 1 was split from the other five sequence groups. The isolates belonging to sequence group 5 gave the highest mean rosetting rate (21.31%). However, within sequence group 2 and group 6, the isolates causing severe malaria had significantly higher rosetting rate than those causing uncomplicated malaria (*p *= 0.014, *p *= 0.007, respectively).

**Conclusion:**

This is the first report of PfEMP1-DBLα analysis in clinical Thai isolates using semi-conserved features (cys/PoLV and PSPBs). The cys/PoLV group 5 gave the highest rosetting rate. PfEMP1-DBLα domains in Thai isolates are highly diverse, however, clinical isolates from severe and uncomplicated malaria shared common sequences.

## Background

Sequestration of PRBC in microvascular endothelium of various organs is the unique property of *Plasmodium falciparum *infection. Cytoadherence and rosetting have been associated with severe disease by blocking blood flow, limiting the local oxygen supply and stimulating cytokine production [1,2]. Intercellular adhesion molecule-1 (ICAM-1) and CD36 are thought to be commonly used receptors [3,4], while the variant *P. falciparum *erythrocyte membrane protein 1 (PfEMP1) expressed on the surface of PRBC is the major parasite ligand [5-7]. *Plasmodium falciparum *isolates from different geographical areas showed variable binding ability to CD36 and ICAM-1 [8,9] and a wide range of ability to form rosettes [10,11], which may contribute to different clinical severity of malaria. Almost all patient isolates bind to CD36 [12], while the binding to ICAM-1 has widely different avidities among clinical isolates [8] and is common among African patients with highest binding in cerebral malaria [13,14]. In laboratory isolates, ICAM-1 binding can be segregated into high and low-avidity binders represented by parasite lines ItG and A4, respectively [15]. Recently, the mutant proteins, ICAM-1^Kilifi ^(a major mutation detected in African populations) [16] and ICAM-1^S22/A ^(alanine scanning mutagenesis of ICAM-1) have been demonstrated to affect the binding ability of variant laboratory parasites A4, ItG and JDP8 in comparison to wide type ICAM-1 (ICAM-1^Ref^) [17]. It is possible that the similar patterns of mutagenesis in domain1 of ICAM-1 affecting the binding of ItG and JDP8 might be linked to their shared high avidity type binding to ICAM-1 [18,19]. A4 had only some critical residues in common with ItG and JDP8 and differed in having a low-avidity ICAM-1 binding phenotype [17]. In fact, cytoadherence of infected RBC to endothelial cells is a multi-step process with various receptors contributing synergistically to promote cell adhesion, such as that seen between ICAM-1 and CD36 in mediating adhesion to endothelium has under static conditions with CD36 meditating the majority of infected RBC [20,21], as well as under flow conditions where the infected RBC appear to tether and roll on ICAM-1, but bind strongly to CD36 [18,22,23]. Likewise, a correlation between severity of malaria and rosetting phenotype could be found in *P. falciparum *isolates from patients in the Gambia, Gabon, and Kenya [10,24,25], while the isolates from patients in Papua New Guinea, Malawi and Thailand could not [9,26-29]. However, the rosetting rate was associated with high parasitaemia in Thai adults [28,30].

PfEMP1 is encoded by the *var *gene family in that the switching between members is associated with changes in antigenicity and binding phenotypes [31-33]. The extracellular portion of PfEMP1 is made up of multiple domains including "Duffy-binding like" (DBL) domains which bind to many host cell receptors according to their sub-types, in which DBLα binds to CR1, CD31 [34] and GAGs implicated in the rosetting phenotype while DBLβC2 binds to ICAM-1. The cysteine-rich interdomain region (CIDR) of PfEMP1 is able to bind to CD36 [35,36]. The study focusing on DBLα emphasizing *var *gene diversity in laboratory and field strains showed that no more than 15–20% of amino acid sequences are conserved in all variants [37]. In laboratory isolate 3D7, six *var *DBLα sequences are diverse with 42–70% amino acid identity [38]. Similarly, the study on genetic diversity of DBLα region in *P. falciparum var *genes among Asia Pacific isolates revealed 46–50% amino acid identity within isolates [39]. However, the conserved regions surrounding hyper-variable regions within DBLα have been reported, which is important in an aspect of conformation or folding for adhesion. The conservation were found in cysteine residue at the position of 8–11 [39] and 16–18 [5,37]. Interestingly, the parasite rosetting phenotype of isolates from Kilifi, Kenya was shown to be strongly associated with the expression of group 2 PfEMP1-DBLα sequences [40]. The transcription of *var *group A and B genes was more abundant in children with clinical malaria than those with asymptomatic infections [41]. Recently, a simple non-phylogenetic network used to visualize the sharing of polymorphic blocks of sequence between large collections of DBLα tags showed that 92% of 1,420 PfEMP1-DBLα sequences from clinical isolates in Kilifi linked together within a single giant component and another sub-group contained sequences found to be associated with the rosetting phonotype [42]. Additionally, the associations between *var *gene expression and severity of malaria have been reported in patients from French Guyana indicating that severe cases are associated with specific *msp1 *allele (B-K1) and specific DBLδ sequences [43], while the lack of 1–2 cysteine residues in PfEMP1-DBLα was associated with severe non-cerebral malaria and rosetting in Brazil [44]. Recently, when the *var *gene transcription in 93 fresh *P. falciparum *trophozoite from Uganda children with severe or uncomplicated malaria through *var*-specific DBL1α-PCR amplification and sequencing was profiled, it has been demonstrated by using a method for sub-sectioning region alignments into homology areas (MOTIFF) that specific PfEMP1-DBL1α amino acid motifs correlated with rosetting and severe malaria, with motif location corresponding to distinct regions of receptor interaction [45]. This study is aimed to demonstrate whether the highly polymorphic PfEMP1-DBLα sequences occurring among Thai isolates causing severe and uncomplicated malaria are associated with their ability to form rosettes and reflect the clinical outcome of the patients. The binding ability to ICAM-1^Ref^, mutant ICAM-1^Kilifi ^and ICAM-1^S22/A ^and CD36 among *P. falciparum *isolates were also evaluated.

## Methods

### Subjects

Seventy nine patients comprising of 49 and 30 patients with severe and uncomplicated falciparum malaria, respectively, admitted to the Hospital for Tropical Diseases, Faculty of Tropical Medicine, Mahidol University, Bangkok, Thailand during the peak of malaria transmission from April 2005 to May 2006 were enrolled with informed consent. The ethical issues of this study have been approved by the Ethical Committee of Faculty of Tropical Medicine, Mahidol University. All patients were positive for *P. falciparum *infection by microscopic examination of thin and thick blood smears stained with Giemsa. Severe and uncomplicated malaria were defined according to World Health Organization criteria [46]. Severe malaria was defined by the presence of asexual forms of *P. falciparum *in the blood with one or more of the following symptoms: cerebral malaria; hyperparasitaemia (> 200,000 parasites/μl); spontaneous bleeding from gum, nose, gastrointestinal tract; repeated generalized convulsion; impaired consciousness; prostration; inability to swallow or retain oral medication; hyperpyrexia; or clinical laboratory tests showing severe anaemia (haematocrit < 15%, haemoglobin < 5 g/dl), renal failure (serum creatinine > 265 μmol/l) or hypoglycaemia (whole blood glucose < 2.2 mmol/l). Malaria patients who lacked these abnormalities were considered as uncomplicated malaria.

### Cultivation of *Plasmodium falciparum *erythrocytic stages

Three milliliters of fresh *P. falciparum *infected blood obtained from patients at admission (day 0) before treatment were immediately cultivated *in vitro *as described [47] in RPMI 1640 medium (Gibco Life Technologies, New York, USA) containing HEPES supplemented with 10% human serum from Thai donors, 2 mM L-glutamine, 2.5 μg/ml gentamicin and 25 mM sodium bicarbonate in a gas mixture of 5% CO_2_, 1% O_2 _in N_2_. Parasite growth was monitored daily by examination of Giemsa-stained thin blood films until the culture reached the pigmented trophozoite stages. At least 1,000 erythrocytes were counted and the parasite stages were recorded and calculated as percent parasitaemia. Parasite adhesion assays, rosette formation and RNA isolation were performed when approximately 70% parasites were trophozoite stages.

### Parasite binding assays

The static protein-binding assays were performed as described previously [48] using purified receptors: ICAM-1^Ref^, ICAM-1^Kilifi ^and ICAM-1^S22/A ^and purified CD36 (R&D Systems, UK). Briefly, three sets of 50 μg/ml ICAM-1 receptor and 10 μg/ml CD36 were spotted onto 35-mm petri dishes (Nunc, Roskilde, Denmark) and incubated at 37°C for 2 hr in a humidified atmosphere. Dishes were blocked with 1% bovine serum albumin in phosphate buffer saline (PBS) overnight at 4°C, washed with RPMI 1640 medium-HEPES and warmed at 37°C for 30 min. The dishes were added with 1.25 ml of each individual *P. falciparum*-infected RBC suspension, which has been adjusted to 3% parasitaemia and 1% haematocrit for binding assay. Assays were incubated at 37°C for 60 min with gentle swirling every 15 min. Dishes were gently washed with RPMI 1640 medium-HEPES until no non-adherent RBCs were visible by inverted microscopy. The bound cells were fixed with 2% glutaraldehyde (Sigma, St Louis, MO) in PBS for 20 min and stained with Giemsa (Merck, Poole, UK). The numbers of bound parasitized red blood cell (PRBC) per mm^2 ^to each receptor were counted under light microscope with oil immersion. The assays were done in duplicate. The binding of more than five PRBCs/mm^2 ^was considered significant, as previously described [9]. The *P. falciparum *laboratory lines, A4 and ItG were used as controls.

### Rosette formation

Rosette formation was performed using 100 μl of fresh PRBC suspension mixed with 2 μl of 0.01% acridine orange solution as previously described [10]. Ten microliters of the suspension were placed under a 22 × 22 mm cover slip and examined under a fluorescence microscope. Two or three hundred trophozoite stages were counted in duplicate. A rosette was scored if 2 or more uninfected RBC were bound to a single infected erythrocyte. The rosette formation was calculated as the ratio of the numbers of rosette to the total number of parasitized red blood cells.

### RNA isolation, RT-PCR and PCR

Total RNA was extracted from malaria culture of 11 and 8 parasite isolates causing severe and uncomplicated malaria, respectively, cultured at least 2 cycles or reaching at least 5% parasitaemia as previously described [49]. RNA was then treated with RNAse free deoxyribonuclease I (Ambion, UK) to degrade the contaminating genomic DNA. Complementary DNA (cDNA) was synthesized by reverse transcribed RNA with cDNA synthesis kits (Bioline, UK) (RT-PCR) using random hexamers according to the manufacturer. For each cDNA synthesis reaction, a control reaction without reverse transcriptase was done with identical amounts of template. 300–400 base pair (bp) fragments of the PfEMP1-DBLα domains were amplified using degenerate primers 2002αAF; 5'-GCA CG(A/C) AGT TT(C/T)GC-3' and αBR; 5'-GCC CAT T(G/C)T CGA ACC A-3' based on those designed previously [50]. The PCR cycling conditions were started with a hot start (93°C for 3 min) followed by 1 cycle of 93°C, 30 sec; 45°C, 30 sec; 72°C, 30 sec, then 29 cycles of 93°C, 30 sec; 45°C, 30 sec; 72°C, 30 sec and extension at 72°C, 10 min. The amplified products were run on 1% agarose gel at 100 volts, stained with ethidium bromide and visualized by UV-transilluminator. The 100 bp marker (Bioline, UK) as standard marker and the DNA of A4 *P. falciparum *strain and the mixture without DNA as positive and negative controls, were used. The expected PCR product at roughly 400 bp was carefully cut from agarose gel and purified using Millipore-Ultrafree^®^-DA (Millipore, UK).

### DNA cloning and sequencing

The purified PCR products were cloned into pCR^®^2.1-TOPO plasmid vectors and used to transform One Shot TOP10 competent cells using TOPO TA Cloning^® ^kit (Invitrogen, USA). The transformed cells were grown overnight and individual white colonies were selected for culture. Plasmids were extracted from overnight cultures using miniprep kit (QIAGEN, Germany) and were sequenced using Beckman CEQ 8000 capillary sequencer (Beckman Coulter, UK). The sequences used in this study were submitted to Genbank (accession numbers: FJ876523–FJ876817).

### Cysteine and position of limited variability (Cys/PoLV) sequence grouping

Sequences selected for analysis were all open reading frames beginning at the position of the 59 consensus motif DIGDI within homology block D and ending at the position of the 39 consensus motif PQYLR within homology block H. The sequences were classified into one of six sequence groups based on a count of the number of cysteine residues within the tag region and a set of sequence motifs at four positions of limited variability (PoLV1–4). The position of limited variability1 (PoLV1) motif was situated at the 3' end of homology block D. The PoLV2 motif was situated at the 5' end of homology block F. The PoLV1 and PoLV4 are fixed in relation to the 5' and 3' ends of the sequence, respectively. The PoLV2 and PoLV3 are fixed in relation to a "WW" motif. Sequence groups were classified as follows: group 1: MFK* motif at PoLV1, 2 cysteines; group 2: *REY motif at PoLV2, 2 cysteines; group 3: 2 cysteines, not group 1 or 2; group 4: 4 cysteines, not group 5; group 5: *REY motif at PoLV2, 4 cysteines; group 6: presence of 1, 3, 5 or 6 cysteines. The asterisk "*" denotes any amino acid. MFK* motifs at PoLV1 and *REY motifs at PoLV2 are mutually exclusive in tag sequences isolated worldwide [51].

### Construction of PfEMP1-DBLα networks

The PfEMP1-DBLα sequence networks were drawn and visualized using the freely available software, Pajek [52]. By Excel spreadsheet (Microsoft) function, four blocks of amino acids from specific windows of DBLα sequence tags defined using three anchor points were extracted from each PfEMP1-DBLα sequence tags designated as position specific polymorphic block (PSPBs). Default positions set for PSPBs were as follows: the 5' amino acid of PSPB1 was set 15 amino acids from the 5' of the tag region; the 3' end of PSPB2 was fixed 5 amino acid 5' to the conserved central WW motif; the 5' end of PSPB3 was fixed at 13 amino acid 3' to the central WW motif and the 3' end of PSPB4 was fixed 13 amino acid from the 5' end of the tag region. The Active Perl program and the script file were used to determine which sequences shared PSPBs and imported into a network analysis package. Each PfEMP1-DBLα sequence represented a vertex within the network. An edge was formed between two vertices if they shared one or more PSPB region. Visualization of the divisions of the sequences into cys/PoLV (positions of limited variability) groups was achieved through formatting the data as a Pajek partition file.

### Data analyses

The rosette formation rate and the binding level of parasitized red blood cells from patients with severe and uncomplicated malaria to ICAM-1 and CD36 were compared by Mann-Whitney U test. The Spearman's correlation test was used to determine the association of ICAM-1 and CD36 binding level. The nucleotide sequences of PfEMP1-DBLα domains were aligned and translated to amino acid using BioEdit computer program. The selected nucleotide sequences containing "homology blocks" of PfEMP1-DBLα [53] were analysed using the specific text string analysis functions in Microsoft Excel and Perl program version 2 developed by Peter Bull [40].

## Results

**Figure 1:**
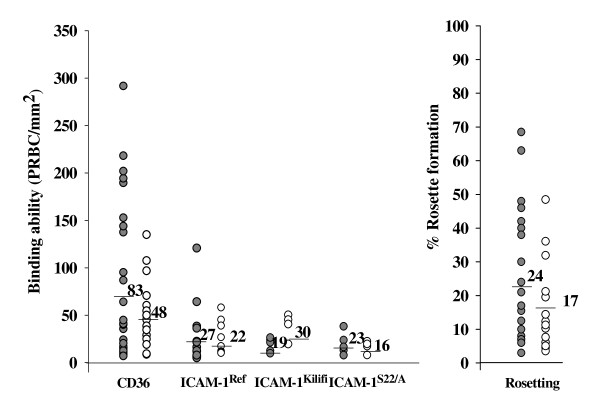
**Binding ability of parasitized red blood cells to CD36 and ICAM-1 and rosette formation**. The binding ability to CD36, ICAM-1^Ref^, ICAM-1^Kilifi^, ICAM-1^S22/A ^(PRBCs/mm^2^) and rosetting rate (%) of isolates causing severe (black dot) and uncomplicated malaria (white dot). The mean number of PRBCs bound to each receptor is shown as a horizontal line. The percent of isolates causing severe (SM) and uncomplicated (UCM) malaria bound to each receptor were shown, CD36: 89% for SM and 78% for UCM; ICAM-1^Ref^: 48% for SM and 43% for UCM; ICAM-1^Kilifi^: 17% for SM and 21% for UCM; ICAM-1^S22/A^: 21% for SM and 17% for UCM; rosette formation: 79% for both SM and UCM.

Among 79 *P. falciparum *isolates from patients, only 52 isolates (29 and 23 isolates causing severe and uncomplicated malaria, respectively) reached mature stage by primary culture sufficient for parasite binding assays. As shown in Figure 1, 48% and 43% of isolates causing severe (SM) malaria and uncomplicated malaria (UCM) bound to wild type ICAM-1 (ICAM-1^Ref^), while the lesser amount of isolates could bind to mutant ICAM-1 proteins (ICAM-1^Kilifi ^and ICAM-1^S22/A^) (ICAM-1^Kilifi^: 17% for SM and 21% for UCM; ICAM-1^S22/A^: 21% for SM and 17% for UCM). However, the binding levels of *P. falciparum *isolates in this study to both wild type and mutant ICAM-1 proteins were relatively low. The assays were validated by using *P. falciparum *laboratory lines A4 and ItG as binding controls [18] which bound to wild type and mutants ICAM-1 and CD36. All isolates that bound to mutant ICAM-1 proteins could bind to wild type ICAM-1 as well. Among isolates that bound to ICAM-1, 40% of isolates causing severe malaria showed major decreases in their binding to both mutant ICAM-1 proteins when compared to their binding levels to ICAM-1^Ref^. However, 37% of isolates causing uncomplicated malaria showed no difference or minor decrease in their binding to ICAM-1^Kilifi ^while 80% showed a major decrease in their binding to ICAM-1^S22/A^. The higher numbers of isolates causing both severe (89%) and uncomplicated malaria (78%) could bind to CD36. Likewise, 79% of isolates both causing severe and uncomplicated malaria were able to form rosettes. Higher numbers of PRBCs could bind to CD36 from isolates causing severe malaria (range = 7–293, mean = 83 PRBC/mm^2^) when compared to those causing uncomplicated malaria (range = 5–120, mean = 48 PRBC/mm^2^), but no significant difference was found (*p *= 0.218). Lower numbers of PRBCs of isolates in both groups of patients bound to ICAM-1^Ref ^(*p *= 0.74), ICAM-1^Kilifi ^(*p *= 0.748) and ICAM-1^S22/A ^(*p *= 0.90) with no significant differences. Similarly, most isolates causing severe disease were able to form rosettes at a higher rate than those causing uncomplicated malaria, but no significant difference was found (*p *= 0.51). Of all isolates tested, 71%and 65% of isolates causing severe and uncomplicated malaria, respectively could bind to more than one receptor. However, when the binding levels to ICAM-1^Ref ^and CD36 were correlated, no significance was observed either among isolates causing severe (*r *= 0.197, *p *= 0.153) or among isolates causing uncomplicated malaria (*r *= 0.309, *p *= 0.076).

**Figure 2:**
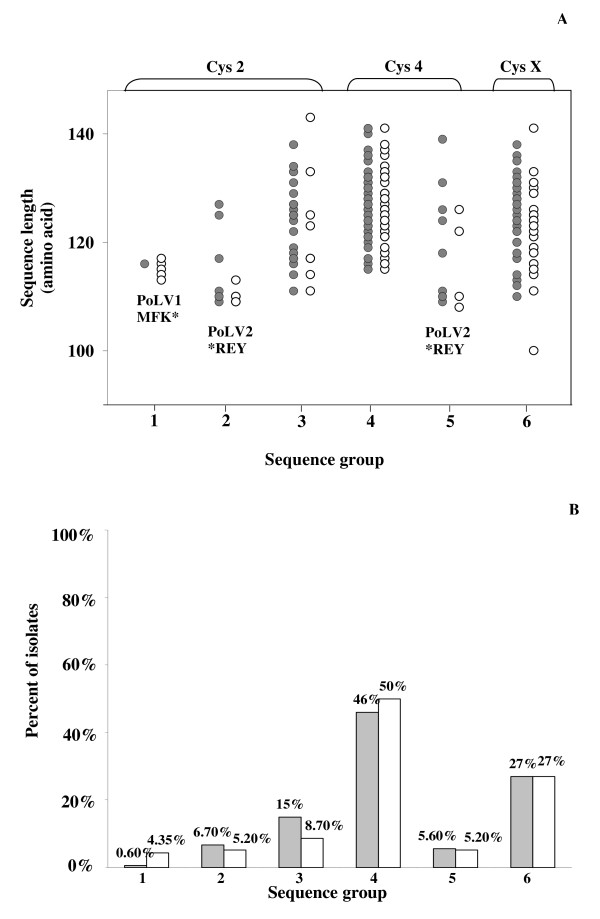
**Sequence groups of PfEMP1-DBLα domains from isolates causing severe and uncomplicated malaria**. (A) Distribution of DBLα sequences of isolates causing severe (black dot) and uncomplicated (white dot) malaria in six sequence groups. DBLα sequences were divided into six sequence groups: sequence groups 1–3 contain two cysteine residues (cys2) and sequence groups 4 and 5 contain four cysteine residues (cys4). Sequence group 6 contains one, three, five, or six cysteines (cysX). Sequence groups 2 and 5 contain PoLV2_*REY_. Sequence group 1 contains PoLV1_MFK*_. The length of each distinct DBLα sequence within each sequence group is indicated; (B) The percent of isolates causing severe (black bar) and uncomplicated malaria (white bar) bound to each receptor.

**Figure 3:**
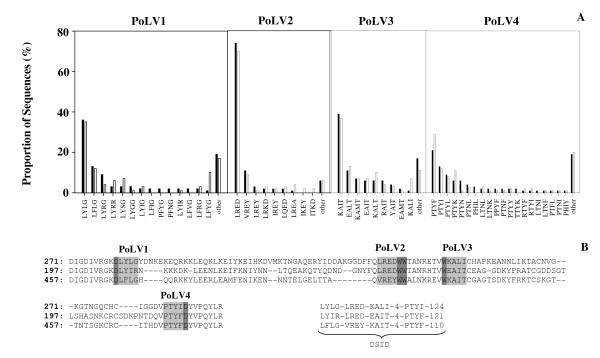
**The semi-conserved regions in PfEMP1-DBLα sequence**. (A) Distribution of PoLV1, PoLV2, PoLV3 and PoLV4 sequences within isolates causing severe (black bar) and uncomplicated malaria (white bar); (B) The location of PoLV1, PoLV2, PoLV3 and PoLV4 in sequence and the distinct sequence identifier (DSID) for each sequence.

Sequencing of the potential rosetting domain, PfEMP1-DBLα was carried out in order to investigate the distribution of PfEMP1-DBLα features between isolates. A total of 295 PfEMP1-DBLα clones were successfully sequenced from randomly selected isolates including 180 sequences from 11 isolates causing severe malaria and 115 sequences from eight isolates causing uncomplicated malaria (Genbank accession numbers: FJ876523–FJ876817). Of these, 281 non-identical amino acid sequences were identified (< 95% sequence identity). When the PfEMP1-DBLα sequences were classified into six sequence groups, designated as sequence group 1–6, using specific sequence features based on a count of number of cysteine residues within the tag region and a set of sequence motifs at four positions of limited variability (PoLV1–4) [40], a similar distribution of sequences in each sequence group was seen between isolates causing severe and uncomplicated malaria (Figure 2). When all six sequence groups of isolates causing severe and uncomplicated malaria were compared using Chi-square, no significant difference was found. However, when individual sequence group causing severe and uncomplicated malaria were compared, only sequence group 1 was significantly different from sequence group 3 (*p *= 0.027). Interestingly, such sequence group 1 were from patients with uncomplicated malaria, while the majority of isolates in sequence group 3 were from patients with severe malaria. The distribution of PoLV1–4 motifs among isolates using a set of sequence motifs at four positions of PoLV1–4 whose positions within the sequence are fixed in relation to four anchor points is shown (Figure 3). PoLV1 and PoLV4 are fixed in relation to the 5' and 3' ends of the sequence, respectively, while PoLV2 and PoLV3 are fixed in relation to a "WW" motif. The majority of sequences in the tested isolates contained PoLV1, PoLV2, PoLV3 and PoLV4 motifs such as "LYLG", "LRED", "KAIT" and "PTYF", respectively (Figure 3A). Likewise, there was a close similarity between the distribution of PoLV1–4 motifs among PfEMP1-DBLα sequences of isolates causing severe and uncomplicated malaria. When the distinct sequence identifiers (DSID) consisting of a string of sequence features in the form of "PoLV1-PoLV2-PoLV3-number of cysteines-PoLV4-sequence tag length" (Figure 3B) are taken into account, there were overall 272 DSIDs. However, no significant difference was found when the number of distinct sequences in isolates causing severe malaria were compared to those causing uncomplicated malaria (*p *= 0.967).

**Figure 4:**
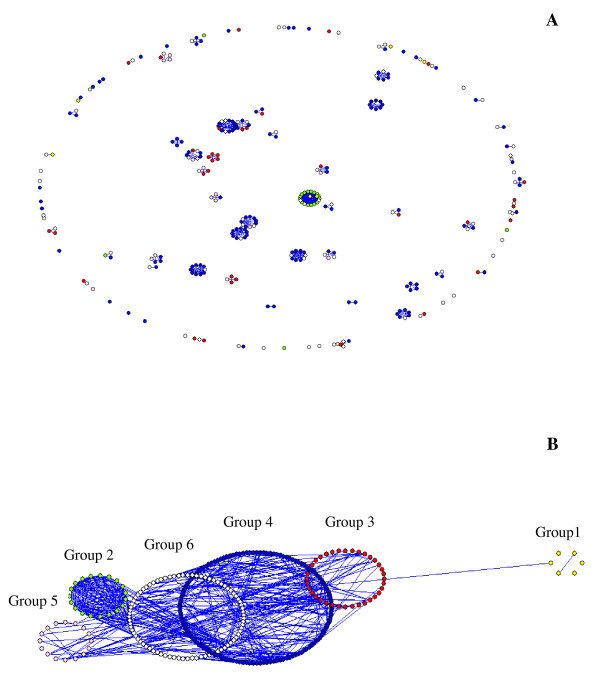
**The structure of a Thai PfEMP1-DBLα sequence network**. (A) The basic network containing 295 sequences constructed by using four position specific polymorphic block (PSPBs); (B) The six sequence group's networks of Thai PfEMP1-DBLα; sequence group 1 (yellow dot), sequence group 2 (green dot), sequence group 3 (red dot), sequence group 4 (blue dot), sequence group 5 (pink dot) and sequence group 6 (white dot).

In order to determine the variation of PfEMP1-DBLα sequences in relation to their ability to form rosette, four levels of rosetting rates were defined as non-rosetting (0%), low rosetting rate (1–19%), moderate rosetting rate (20–39%) and high rosetting rate (> 40%). The distribution of PfEMP1-DBLα in six sequence groups showed significant associations with rosetting levels (*p *= 0.028). To understand the relationship between 295 PfEMP1-DBLα sequences from Thai clinical isolates, a simple non-phylogenetic approach to visualize the sharing of polymorphic blocks (position specific polymorphic block; PSPB) of sequences was employed. The PSPBs networks are represented by nodes (vertices) that are joined by lines if they are identical at one or more their constituent PSPBs [42]. However, as shown in Figure 4A, the PSPBs networks could not differentiate severe from uncomplicated malaria. Few unbroken networks of PfEMP1-DBLα sequences were seen. The network was constructed as shown in Figure 4B based on the six sequence groups classified by PoLV1–4 and together with the numbers of cysteine residues within the PfEMP1-DBLα sequences. The main component of the PSPBs network consists of the sequence groups 2, 3, 4, 5 and 6 while sequence group 1 was splitting with no connection. Almost all PfEMP1-DBLα sequences in group 1 were from uncomplicated malaria and gave low rosetting rates (mean = 3.6%). In contrast, the isolates belonging to sequence group 5 gave the highest mean rosetting rate (21.3%) and in sequence groups 2 and 6, the isolates causing severe malaria had significantly higher rosetting rates than those causing uncomplicated malaria (*p *= 0.014, *p *= 0.007, respectively).

## Discussion

*Plasmodium falciparum *isolates show different abilities to form rosettes and binding to CD36 and ICAM-1 which may contribute to different clinical severity of malaria [1,2]. In this study isolates from both severe and uncomplicated malaria in Thailand could bind to CD36 while only 45% could bind to wild type ICAM-1 and at lower levels than to CD36. This is similar to a previous study that almost all *P. falciparum *isolates from Thai patients could bind to CD36 while only a few isolates (13%) could bind to ICAM-1 [54]. Furthermore, CD36 binding was identical in parasites from cerebral malaria patients and community controls (selecting on the basis of those who had *P. falciparum *parasitaemia), but slightly elevated in non severe cases in Kilifi [8]. It has been reported that the ability to bind to ICAM-1 showed widely different avidities in clinical isolates [8]. In this study, a lower percentage of isolates could bind to ICAM-1 while at least 80% of isolates from Kenyan patients could [8,55] indicating that the *in vitro *binding ability to ICAM-1 among the isolates from different geographical endemic areas seems to be variable.

It has been reported that rosette formation was associated with severity of malaria [24] such as severe anaemia [8,9,55] and cerebral malaria in children [10,25]. However, 79% of isolates causing both severe and uncomplicated malaria in our study could form rosettes at a similar rate. In general, rosetting seems to increase microvascular obstruction of the blood flow [56] and may hide the infected cell thereby protecting it from phagocytosis, one of the main mechanisms of anti-parasitic immunity [57]. Therefore, it could be concluded at this point that cytoadherence to receptors expressed on endothelial cells resulting the focal accumulation of parasitesat high densities leading to microvascular obstruction would restrict the exchange of glucose and oxygen at the capillary level which together with hypoglycaemia, lactic acidosis, high grade of fever and high TNF level might lead to representing the mechanism of disease [58].

Since PfEMP1-DBLα is the rosetting domain, the sequence was further investigated to see whether there was any association with their rosetting rates. PfEMP1-DBLα domains from both severe and uncomplicated malaria are highly variable in length and amino acid sequences. The distributions of the position of limited variability (PoLV1–4) and sequence groups among isolates causing severe and uncomplicated malaria were similar, indicating that PfEMP1-DBLα shared common sequence among different clinical categories. When the sequence networks were further constructed based on the position specific polymorphic block, PSPB and cys/PoLV, the results showed that the main component of PSPBs network consisted of almost all sequence groups which supports the common sequence phenomenon. However, sequence group 1 was split from the main component indicating different sequences from the other five groups. It is possible that the different sequences in group 1 may be associated with less severe malaria since the isolates originated from patients with uncomplicated malaria and had low rosetting rates. The majority of isolates from severe malaria with the highest rosetting rates belonged to sequence group 5. Additionally, there was an association between sequence group 2 and rosette formation of isolates with high rosette rates from severe malaria cases, but not with low rosette rates and uncomplicated malaria. This is in accordance with the study in Kilifi children demonstrating that sequence group 2 expression is positively associated with the percentage of infected red blood cells that formed rosettes [40]. However, the results were different from those demonstrated in African isolates where rosette frequency of isolates from adults in Mali was significantly correlated with group 1 genes, but not with any other group and two cysteine residues sequences, which were more frequent among isolates from children with cerebral malaria than those with hyperparasitaemia [59]. It seems to be that the networks in our study were different from the Kilifi network in that few unbroken networks were seen in the Kilifi network with two apparent major lobes, one small and the other large [42]. This might be possibly due to the different sample sizes used in these two studies as well as the different populations in which the Kilifi network were analysed from children whereas the Thai network in this study was carried out in adults.

The variation in *in vitro *binding ability to CD36 and ICAM-1, the ability to form rosettes and in PfEMP1-DBLα sequences among clinical isolates might be due to (i) severe malaria includes a variety of clinical syndromes e.g. severe anaemia, cerebral malaria, respiratory distress, which may have different underlying pathogenic mechanisms [58], (ii) the tested clinical isolates originated from different geographical malaria endemic areas (iii) the PfEMP1-DBLα sequence groups which showed association with rosette phenotype were different among isolates of different geographic areas.

## Conclusion

This is the first report on the connection between PfEMP1-DBLα sequences in Thai isolates causing severe and uncomplicated malaria using the software designed for analysis of social networks based on small blocks of the semi-conserved regions (PoLVs and/or PSPBs) in relation to rosette formation. The cys/PoLV group 5 gave the highest rosetting rate. The sequence group 1 was split from the other 5 sequence groups by using a simple non-phylogenetic approach. However, a larger sample size and isolates from different geographical areas should be further investigated. The level of *var *gene transcription should be determined in order to draw a definite conclusion.

## Consent

The present study was approved by the Ethical Committee of Faculty of Tropical Medicine, Mahidol University.

## List of abbreviations used

ICAM-1: intercellular adhesion molecule-1; (P)RBC: (parasitized) red blood cell; PfEMP1: *Plasmodium falciparum *erythrocyte membrane protein 1; DBL: Duffy binding like.

## Competing interests

The authors declare that they have no competing interests.

## Authors' contributions

SK and AC designed the study, SK, AC and TK, were responsible for the supervision of the work, AC assisted in PCR and sequencing, AC and NH were responsible for the sequences analysis, SK, AC and NH drafted the manuscript. All authors read and approved the final manuscript.
